# Effect of Mandible Phantom Inclination in the Axial Plane on Image Quality in the Presence of Implant Using Cone-Beam Computer Tomography

**DOI:** 10.7759/cureus.36630

**Published:** 2023-03-24

**Authors:** Sonam Khurana, Pranav Parasher, Adriana G Creanga, Hassem Geha

**Affiliations:** 1 Oral Pathology, Radiology, and Medicine, New York University College of Dentistry, New York City, USA; 2 Diagnostic Radiology, Rutgers School of Dental Medicine, Newark, USA; 3 Oral and Maxillofacial Radiology, Rutgers School of Dental Medicine, Newark, USA; 4 Oral and Maxillofacial Radiology, University of Texas Health Science Center at San Antonio, San Antonio, USA

**Keywords:** beam hardening artefacts, oral implants, contrast to noise ratio, artifact, cone- beam computed tomography

## Abstract

Purpose

To assess the effect of 30° phantom inclination on image quality in the presence of an implant using cone-beam computed tomography (CBCT).

Materials and methods

Three series of eight scans were taken and categorized by a range of 87-90 kVp and 7.1 mA, and 8 mA. For the first CBCT series, the phantom was placed on a flat plane. For the second series, the phantom was inclined at 30° in the axial plane. For the third series, inclined scans were re-oriented and included for statistics. In total, 24 scans were used for statistics. i.e., eight scans at three different planes (flat plane, inclined plane, and re-oriented inclined plane). All the images were analyzed for artifact and contrast-to-noise ratio (CNR) on ImageJ software.

Results

The inclination of the dry human mandible phantom by 30° reduces the artifact (p <0.05). However, the CNR was not affected by the phantom inclination.

Conclusion

The appropriate inclination of the head can significantly reduce the metal artifact in the presence of implants and thus improve the CBCT image quality for post-operative follow-up.

## Introduction

An artifact is a feature on the reconstructed image that is not characteristic of the subject under study. Cone-beam computed tomography (CBCT) units inherently produce artifacts due to the inconsistency between the technical configurations of the scanner and the back-projection reconstruction methods [1]. Metal artifacts limit the use of CBCT for post-operative implant evaluation. Metallic objects affect the reconstructed image quality due to beam hardening, scatter, and photon starvation. Beam hardening is caused by the preferential absorption of low-energy X-rays by metal objects resulting in increased mean beam energy [2]. Beam hardening appears as dark streaks on the reconstructed image [1]. The deflection of X-rays from their original path due to interaction with different objects is known as the scatter. Scatter results in faulty projection data and produces dark streaks in the reconstructed images, like beam hardening [3]. Photon starvation is the complete absorption of photons along the beam path. These effects can result in image deterioration ranging from bright streaking and dark areas adjacent to metallic structures to complete loss of gray values [2]. Beam hardening artifact causes gray values variations around the metallic object and decreases the contrast resolution of the image [4]. Contrast resolution is defined as the ability to differentiate minor attenuation differences of tissue and display them with different gray values [3]. The entity that quantifies a system's ability to distinguish between structures and noise in an acquired image is the contrast-to-noise ratio (CNR) [5]. CNR has been more closely related to image quality than image noise [6]. Many studies have been documented in the literature to reduce metal artifacts. Various factors like exposure parameters, size of the field of view (FOV), the central or peripheral position of the subject within the FOV, reconstruction algorithm, and pre-and post-processing techniques have been explored to reduce metal artifacts [7-11]. The increase in Kilovoltage peak (kVp) increases the mean and maximum energy of the photon and reduces the low-energy photons. It makes the implant more and more transparent for high-energy photons. The one disadvantage of high kVp is reduced subject contrast. Therefore, longer grayscale of contrast reduces the overall image quality. The increase in mA and exposure time directly increases the number of photons and decreases the quantum noise, improving the image quality. The improved image quality is limited due to proportionally increased patient dose. A smaller FOV is desirable because it reduces the scattered radiation and unnecessary exposure to the patient. However, it primarily depends on individual needs. In some clinical situations, it is challenging to avoid beam hardening artifacts from the ipsilateral/contralateral side of the arch. Few other studies investigated the effect of tilting the subject or gantry in multidetector CT (MDCT) [12]. During CBCT scan acquisition, the patient is placed in the Frankfort plane. In the Frankfort plane, the patient's head orientation is such that it orients the X-ray source, teeth, implant, and detector in a single plane [13]. The single-plane arrangement causes the high X-ray absorbing components to be arranged in a line. Hence, this study hypothesizes that phantom inclination decreases the metal artifact and CNR. The effect on image quality becomes more evident with multiple implants because titling inhibits the overlapping of high X-ray-absorbing components. Therefore, the analysis of metal artifact reduction after subject tilting can be investigated [13,14]. This in vitro study aimed to evaluate how phantom titling affects the artifact on the right and left sides of the mandible in the presence of two implants oriented parallel to the vertical axis of the bone in the right and left premolar regions. The study also aimed to evaluate the effect of phantom inclination on CNR.

## Materials and methods

Power analysis

We did a power analysis at a power of 0.8 (alpha = 0.05). The power analysis indicated that to detect the difference between study groups (flat plane, inclined plane, and re-oriented inclined plane), two similar implants can be used (titanium grade 4 implants) at the right and left premolar site at eight different combinations

Image phantom

In a partially dentate human dry mandible, two titanium implants were placed in the second premolar region, one on each side. The dry mandible was attached on a flat surface, and two epoxy resin-based substitute (ERBS) blocks were attached. One block (soft tissue equivalent) was attached to the dry mandible in the right buccal canine-premolar region close to the implant. The second block (bone equivalent) was placed on a flat surface parallel to the left lingual ramus-molar area, which served as the control. The phantom was placed in a container filled with water and put on a CBCT machine platform.

Image acquisition

**Figure 1:**
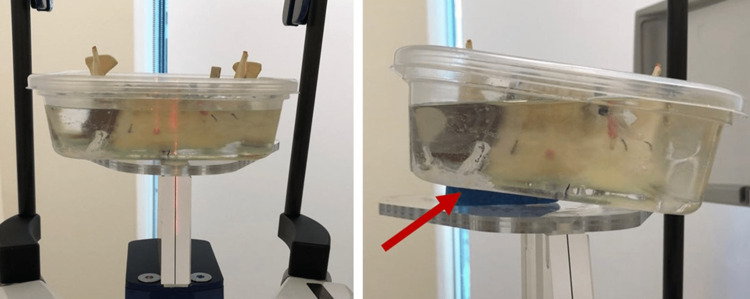
A) Arrangement of the phantom at flat plane and B) 30◦ inclination as marked by red arrow.

For CBCT scans, the Planmeca CBCT machine (Promax 3D max; Planmeca, Helsinki, Finland) was used with a 10 cm x 16 cm FOV and voxel size of 0.4 mm. The coronoid process was chosen as a stable landmark to guide re-orientation. The smaller voxel size of 0.2 mm helped increase the spatial resolution and the quantum noise. To avoid the noisier image, 0.4 mm was finally considered. The implants were placed in the middle of the FOV during all scans using laser positioning beams for reproducibility. Two series of eight scans were taken. For the first CBCT series, the phantom was placed on a flat plane (0° inclination) (Figure 1A) in the center of the field. For the second series, the phantom was inclined at 30° (Figure 1B) in the axial plane towards the left side using a custom wedge in the center of the field.

After inclination, the right side moved up and the left side down. Each series was divided into eight groups of different combinations. The combinations were made at 87 kVp, 88 kVp, 89 kVp, and 90 kVp at 7.1 mA and 87 kVp, 88 kVp, 89 kVp, and 90 kVp at 8 mA. The effect of kVp and mA on artifact and CNR was evaluated separately using the Chi-square test. After the Chi-square test, the groups considered for the study showed no statistically significant relation between closely varying kVp and mA with artifact and CNR individually. The artifact was assessed for kVp and mA on eight axial sections of eight scans at the flat plane individually for the right and left sides. The CNR was evaluated for kVp and mA on 16 axial sections of eight scans at the flat plane.

Evaluation of re-oriented scans

**Figure 2:**
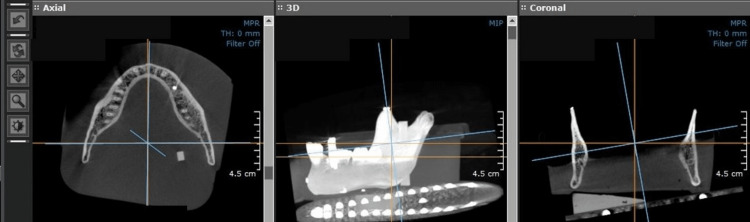
Re-orientation of the scan.

The re-oriented scans were derived from the inclined plane. For the re-oriented scans (Figure 2), high-density markers were placed bilaterally on the top of the coronoid processes. The markers were used as a guide for re-orientation using the "Axis and Re-slice" option on On-Demand software. First, the markers were oriented parallel to each other in the coronal plane and simultaneously observed to overlap in the sagittal plane. The sagittal plane, unlike axial and coronal planes, appears as maximum intensity projection (MIP) rather than multiplanar reformation (MPR) on the "Axis and Re-slice" option on On-Demand software. The final re-orientation was confirmed on the axial plane on two sections by a line passing through a) the same markers placed on the coronoid processes and b) the bilateral posterior ramus region. The re-orientation was performed to realign anatomic structures symmetrically by taking advantage of the isotropic volumetric data set of CBCT. In total, 24 scans were used for statistics. i.e., eight scans at three different planes (flat plane, inclined plane, and re-oriented plane).

Scan evaluation

The volumes were exported in the digital imaging in communications in medicine (DICOM) format to public domain image analysis software, ImageJ (NIH Image, Bethesda, MD; available from rsb.info.nih.gov/ij/). All images were viewed and evaluated in a dimly lit room on a computer monitor (Dell U2410 ultrasharp, 23 inches, resolution 1920×1200; Dell, Round Rock, TX, USA).

Artifact evaluation

**Figure 3:**
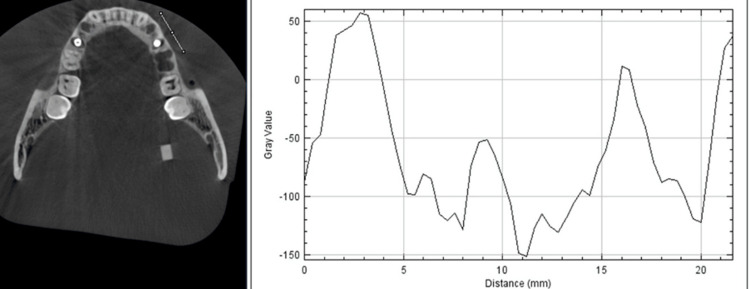
Profile line on ImageJ software to measure the artifact.

An axial section was chosen for the flat, re-oriented, and inclined planes to evaluate artifacts generated by the implants on each side. The section was oriented seven mm (seven axial slices of 1 mm thickness each) superior to the implant apex. For each volume, sections were selected at a similar distance (based on orientation) from the apex, and DICOMS were imported to Image J software. A 15 mm line was plotted 3 mm from the implant, crossing through beam hardening and streaking to evaluate the artifact (Figure 3). The line was plotted bilaterally parallel to the buccal cortex of the dry mandible. Three macros were created for the flat, re-oriented, and inclined planes (one for each) and applied to all the selected images to standardize the profile lines. After applying the macro, the "Plot Profile" option on 'Image J' was used. The difference between the highest and lowest gray values was calculated and noted as an artifact on the plotted profile. A total of 24 axial sections were evaluated for the artifact, one on each scan.

CNR evaluation

**Figure 4:**
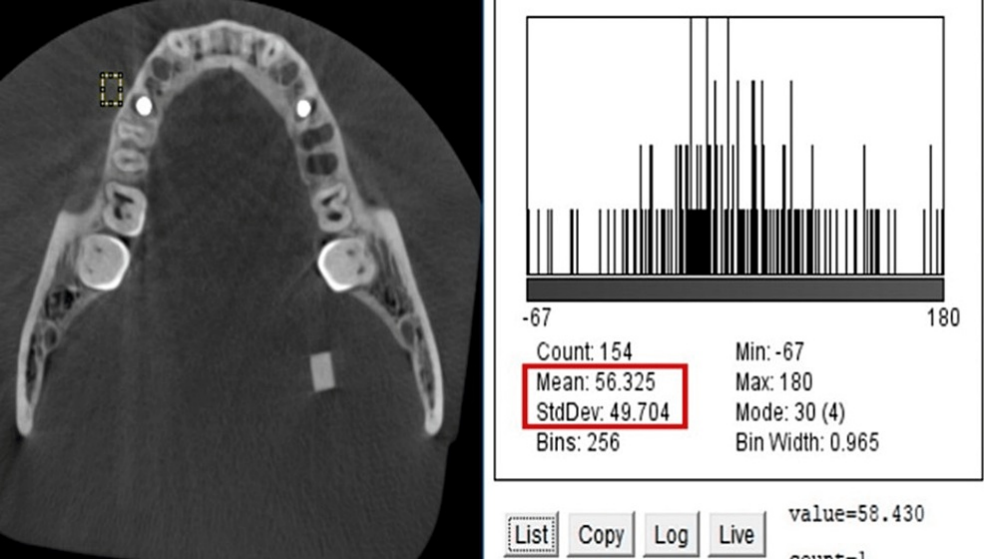
Histogram generated on soft tissue equivalent ERBS block close to the implant. ERBS: Epoxy resin-based substitute.

**Figure 5:**
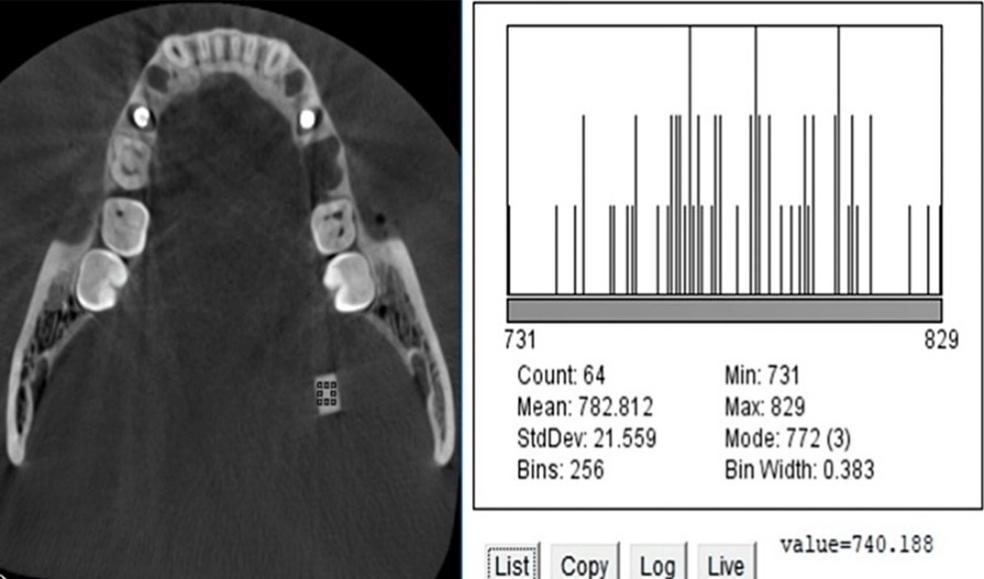
Histogram generated on bone equivalent ERBS block (control) away from the implant. ERBS: Epoxy resin-based substitute.

One axial section was chosen for the flat plane, re-oriented plane, and inclined plane volume. The section was oriented seven mm (seven axial sections of 1mm thickness each) superior to the implant apex. Another axial section, five mm (five sections of 1mm thickness each) inferior to the apex of the implant where the implant was not visible, was chosen separately for the flat plane, re-oriented plane, and inclined plane volumes. Forty-eight sections were evaluated, two on each volume, and DICOMS were imported to the Image J software. A macro was created and applied to all the images to maintain reproducibility. On each measured slice, the first histogram (Figure 4) was acquired on the ERBS block close to the implant, and another histogram (Figure 5) was acquired on the control ERBS block attached away from the implant in the molar-ramus area. The mean gray value and SD were computed from the area histograms. The CNR was calculated as the difference between the mean ERBS and control gray values divided by the SD difference.

Data analysis

One oral and maxillofacial radiologist with experience of more than seven years evaluated the images. The observer was trained and calibrated to perform the measurements on ImageJ software. All measurements were performed twice by the same observer in three weeks intervals. Cronbach's Alpha was used to check for intra-observer variability for artifact and CNR measurements. The Chi-square test was used to evaluate the effect of the change of kVp and mA on artifact and CNR. One-way ANOVA was also used to compare the difference between three groups (flat plane, re-oriented plane, and inclined plane) for artifact and CNR measurements. Post-hoc Bonferroni test was used for multiple comparisons (flat vs. inclined, flat vs. re-oriented, inclined vs. re-oriented) between three groups for artifact and CNR values. The significance for all tests was established at P < 0.05.

## Results

The intraclass correlation using Cronbach's Alpha measurements showed excellent reproducibility for both artifact and CNR. The value for the intraclass agreement was 0.993 and 0.965, respectively, for artifact and CNR.

Effect of kVp and mA

The Chi-square test was used to evaluate the effect of kVp and mA on artifact and CNR (Tables 1-2). The kVp and mA using the Chi-square test revealed no statistically significant difference for both artifact and CNR.

**Table 1:** Effect of kVp on CNR. CNR: Contrast-to-Noise ratio.

Variables	At 7.1 mAs		At 8 mAs	
	kVp	CNR value	kVp	CNR value
CNR close to implant	87	19.22	87	19.31
	88	19.89	88	19.54
	89	19.11	89	20.89
	90	20.76	90	20.32
P-value	>0.05		>0.05	
CNR 5 slices away from implant	87	23.31	87	23.64
	88	23.39	88	24.48
	89	24.56	89	24.93
	90	24.78	90	25.31
P-value	>0.05		>0.05	

**Table 2:** Effect of kVp on artifact for the right and left side implants.

Variables	At 7.1 mAs		At 8 mAs	
	kVp	Artifact value	kVp	Artifact value
Artifact on right side	87	258.47	87	252.84
	88	253.91	88	247.67
	89	252.34	89	243.34
	90	251.81	90	239.30
P-value	>0.05		>0.05	
Artifact on left side	87	259.91	87	247.67
	88	258.47	88	243.34
	89	257.81	89	242.83
	90	256.34	90	242.31
P-value	>0.05		>0.05	

Artifact

The average values of artifact measurements for three different groups on both sides (flat plane, re-oriented plane, and inclined plane) are presented in Table 3. The mean of the calculated artifact on the right side at the flat plane was 249.96, the inclined plane was 203.05, and the re-oriented plane was 193.21. On the left side, the mean of the calculated artifact for the flat plane was 250.91, the inclined plane was 233.36, and the re-oriented plane was 230.64. The re-oriented plane showed the lowest artifact on both sides, followed by the inclined plane. The highest artifacts were produced on the flat plane. One-way ANOVA showed a statistically significant difference on the right side (p <0.05) and no difference on the left side (p >0.05).

**Table 3:** Artifact measurements (as the average difference in gray values) for the right and left side implants for three groups (flat plane, re-oriented plane, and inclined plane). * represents statistically significant values.

Variables	Groups	Mean	P-value
Artifact on the right side	Flat plane	249.96	<0.05*
	Inclined plane	203.05	
	Re-oriented plane	193.21	
Artifact on the left side	Flat plane	250.91	>0.05
	Inclined plane	233.36	
	Re-oriented plane	230.64	

The values for multiple intergroup comparisons using the posthoc Bonferroni test are presented in Table 4. On the right side, the artifact values showed a statistically significant difference (p<0.05) between the flat vs. re-oriented plane and the flat vs. inclined plane. There was no statistically significant difference between the inclined and re-oriented planes on the right side. No statistical difference was noted on the left side between all three groups. 

**Table 4:** Artifact measurements (as the average difference in gray values) for multiple intergroup comparisons using the posthoc Bonferroni test. * represents statistically significant values .

Variables	Groups	Mean Difference	P-value
Artifact on right side	Flat vs. Inclined	46.91	<0.05*
	Flat vs. Re-oriented	56.75	<0.05*
	Inclined vs. Re-oriented	9.84	>0.05
Artifact on left side	Flat vs. Inclined	17.55	>0.05
	Flat vs. Re-oriented	20.27	>0.05
	Inclined vs. Re-oriented	2.72	>0.05

CNR

The average values of CNR measurements for both levels (close to and away from the implant) for three different groups (flat plane, re-oriented plane, and inclined plane) are presented in Table 5. The mean of the calculated CNR close to implant at the flat plane was 19.88, the inclined plane was 23.76, and the re-oriented plane was 25.25. CNR five sections away from the implant, the mean of the calculated artifact for the flat plane was 24.30, inclined plane 24.52, and re-oriented plane 24.96. The re-oriented plane showed the highest CNR at both levels, followed by the inclined plane. The CNR showed the lowest value on the flat plane. However, one-way ANOVA did not show a statistically significant difference for CNR at both levels' flat, inclined, and re-oriented planes (p-value > 0.05).

**Table 5:** Mean values of the CNR for three groups (flat plane, reoriented plane, and inclined plane). CNR: Contrast-to-Noise (CNR) ratio.

Variables	Groups	Mean	P-value
CNR close to implant	Flat plane	19.88	>0.05
	Inclined plane	23.76	
	Re-oriented plane	25.25	
CNR 5 slices away from implant	Flat plane	24.30	>0.05
	Inclined plane	24.52	
	Re-oriented plane	24.96	

The values for multiple intergroup comparisons using the posthoc Bonferroni test are presented in Table 6. No statistically significant difference (p-value > 0.05) was noted between flat vs. re-oriented plane, flat vs. inclined plane, and inclined vs. re-oriented at both levels.

**Table 6:** CNR values for intergroup multiple comparisons using posthoc Bonferroni test. CNR: Contrast-to-Noise ratio.

Variables	Groups	Mean Difference	P-value
CNR close to implant	Flat vs. Inclined	3.88	>0.05
	Flat vs. Re-oriented	5.37	>0.05
	Inclined vs. Re-oriented	1.49	>0.05
CNR 5 slices away from implant	Flat vs. Inclined	0.22	>0.05
	Flat vs. Re-oriented	0.66	>0.05
	Inclined vs. Re-oriented	0.44	>0.05

## Discussion

A radiographic assessment of the proposed implant site is essential to evaluate the bone quality and surrounding vital structures [15]. CBCT is the current method for the preoperative phase whenever cross-sectional imaging is required. In a follow-up examination using CBCT, the implant and associated metal artifacts obscure the evaluation of peri-implant bone osteointegration [16]. Many methods have been used in the literature to reduce metal artifacts [7-11]. Our study focuses on the inclination of the phantom to reduce artifacts and improve image quality. Unlike previous inclination studies [13,14], we used a dry human mandible phantom to simulate the clinical condition. The normal physiological bone can cause radiation scattering and cannot be assessed from a homogenous phantom [17]. In CBCT, the noise changes as a function of kV, mA, basis images, slice thickness, and voxel size [11,18,19]. Our study kept the basis images, slice thickness, and voxel size constant. kVp and mA were varied and showed no statistically significant difference for the artifact and CNR. Our results were different from Pauwels R et al. study [18]. In their study, CNR improved with the increase in kVp and mA because, in that study, kVp and mA varied from 60 to 90 and 1 to 8, respectively. However, our study varied over a small range, i.e., 87 to 90 kVp and 7.1 and 8 mA. An increase in kVp reduces the beam hardening artifact because penetration through metallic objects increases. High kVp and mA increase the CNR attributed to the increased number of photons that reduce the quantum noise. The contrast part of CNR is relatively fixed because it is independent of the exposure parameters. Due to the close variation of the kVp and mA in our study, mean energy did not increase to a great extent to influence artifact and quantum noise, and kVp, ranging from 87 to 90, is already in the higher range. In CBCT, due to the arrangement of all image formation components in the single plane, the inclination causes the spatial redistribution of the artifact [13]. Our study showed a significant reduction in artifacts after the inclination of the phantom toward the left side. Our results were consistent with Min CK amd Kim KA study [14], although their methodology differed. In their study, there were three rotation angles, i.e., alpha, beta, and gamma, and two implants were adjacent. In their research, the artifact was reduced (∆GV was increased) in the inter-implant area due to increased alpha angle rotation. The beta and gamma angles showed no significant change in gray value. Due to the placement of the implant next to each other, forward titling (alpha rotation) resulted in the reduced artifact. In our study, implants were oriented in the right and left quadrants in the premolar region. The inclination towards the left side resulted in the reduced artifact. To conclude, both studies showed artifact reduction due to inclination that primarily depends upon the implant orientation. The result of our study was in accordance with Luckow M et al.'s study [13]. The methodology of that study included varying accelerating voltage, beam current, the starting rotation angle of the mandible in the source-detector plane, and the tilt angles of the jaw with respect to the source-detector plane. The rotation was performed in frontal and sagittal planes. Their study indicated that tilting the mandible by about 14◦ improves the image quality by almost a factor of two (more in the frontal plane than the sagittal plane). Their study showed more improvement in the frontal plane because implants were oriented next to each other. As explained earlier, the orientation of implants was different in our study, and both implants were oriented on the right and left sides of the mandible. Therefore, in our study, rotation was performed towards the left, resulting in a reduced artifact.

The re-oriented scans were assessed separately for artifact and CNR evaluation. The re-oriented scans consistently showed less artifact and better CNR close to the implant. It is because re-oriented scans were derived from the inclined plane. The improvement in artifact and CNR is due to the rearrangement of slices. It also helps to orient the scan in the symmetric anatomic relationship. The CNR is the most used parameter to assess image quality. Phantom inclination did not show statistically significant improvement for CNR; however, near the implant, it consistently improved for inclined and re-oriented planes more than the flat plane. The CNR improvement could be attributed to the spatial redistribution of artifacts and the improvement of associated gray values. Luckow M et al. [13] and Min CK and Kim KA study [14] used different angles and planes to evaluate the results. Although the present study used a different orientation of implants (right and left side of the mandible in the premolar region), the limitation of the study is the use of one inclination direction (left side) and one angle, i.e., 30◦. Further research could be done to allow the operator to use different angles and planes and select the optimum inclination angle of the phantom for the source-detector plane. Vadiati Saberi B et al. [20] performed a quantitative analysis to assess the effect of slice inclination and object position in the central and peripheral portions of the FOV. The authors grouped four types of images: central/ cross-sectional, central/coronal, peripheral/cross-sectional, and peripheral/coronal. They measured the buccolingual dimension (width) and alveolar crest to the bone's inferior border of the mandible dimension (height). The width of the incisor and premolar areas and the height of the incisor, canine, and molar areas showed statistically significant differences in the peripheral/coronal images compared to the direct measurements. Molar area height in the central/coronal slices differed significantly from the direct measurements. Our study showed qualitative analysis, whereas the study by Vadiati Saberi B et al. [20] performed quantitative results. Both studies showed statistically significant results of inclination. Swartz EE et al. [21] stated that the mean range of neck rotation is up to 90◦ on both sides. We inclined our phantom to 30◦, i.e., one-third of maximum rotation. While selecting 30◦ phantom inclination, we assumed two clinical factors to help the CBCT operator simulate the study in the clinical setting. Two factors are 1) To avoid neck muscle fatigue and associated patient movement during the scan, and 2) The size of the gantry will not allow its smooth rotation without interference if the patient's head is maximally rotated.

## Conclusions

This study suggested that 30◦ phantom inclination towards the left side resulted in artifact reduction when two implants were oriented in the right and left premolar regions. The result of this study did not show any improvement in the CNR. The results can be used to simulate clinical research to reduce artifacts and improve CBCT images.
